# Ultra-low thermal conductivities in large-area Si-Ge nanomeshes for thermoelectric applications

**DOI:** 10.1038/srep32778

**Published:** 2016-09-21

**Authors:** Jaime Andres Perez-Taborda, Miguel Muñoz Rojo, Jon Maiz, Neophytos Neophytou, Marisol Martin-Gonzalez

**Affiliations:** 1 Instituto de Microelectrónica de Madrid (IMM-CSIC), Calle de Isaac Newton 8, Tres Cantos, 28760 Madrid, Spain; 2 School of Engineering, University of Warwick, Coventry, CV4 7AL, UK

## Abstract

In this work, we measure the thermal and thermoelectric properties of large-area Si_0.8_Ge_0.2_ nano-meshed films fabricated by DC sputtering of Si_0.8_Ge_0.2_ on highly ordered porous alumina matrices. The Si_0.8_Ge_0.2_ film replicated the porous alumina structure resulting in nano-meshed films. Very good control of the nanomesh geometrical features (pore diameter, pitch, neck) was achieved through the alumina template, with pore diameters ranging from 294 ± 5nm down to 31 ± 4 nm. The method we developed is able to provide large areas of nano-meshes in a simple and reproducible way, being easily scalable for industrial applications. Most importantly, the thermal conductivity of the films was reduced as the diameter of the porous became smaller to values that varied from *κ* = 1.54 ± 0.27 W K^−1^m^−1^, down to the ultra-low *κ* = 0.55 ± 0.10 W K^−1^m^−1^ value. The latter is well below the amorphous limit, while the Seebeck coefficient and electrical conductivity of the material were retained. These properties, together with our large area fabrication approach, can provide an important route towards achieving high conversion efficiency, large area, and high scalable thermoelectric materials.

Silicon based materials and alloys have been successfully used to satisfy the latest technological challenges of our society^1^,^2^. Silicon has several advantages, such as a low cost, abundance, non-toxic properties and easy industrial scalability. Silicon and Germanium present distinct and interesting transport properties. However, composites made of silicon-germanium (Si-Ge) have resulted in an improvement in terms of their transport properties. Currently, these alloys are used in different applications, such as microelectronic devices and integrated circuits, photovoltaic cells, and thermoelectric applications^3^,^4^,^5^. With respect to thermoelectricity, in the last decades Si-Ge has attracted significant attention as an energy harvesting material, for powering space applications^6^,^7^,^8^. Thermoelectric materials transform heat into electricity, and vice-versa, by means of the Seebeck and Peltier effects^9^,^10^. However, the use of these materials for energy harvesting is limited by their poor efficiency and high prices in comparison to other technologies. This efficiency is quantified by the dimensionless figure of merit, 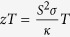, which depends on the electrical conductivity (σ), Seebeck coefficient (*S*) and the thermal conductivity (*κ*) of the material at a temperature *T*^10^,^11^. Two different approaches are commonly used to increase the efficiency of those materials: *i*) the enhancement of the power factor (*S*^2^*σ*) or/and *ii*) the reduction of the thermal conductivity (*κ*), while trying not to alter the other fundamental transport properties of the material. Generally, not only for Si-Ge films but also for different materials, the second approach is the one employed the most. Nanostructuring has been proven theoretically and experimentally to be a successful way to reduce significantly the thermal conductivity of materials^2^,^12^,^13^,^14^. A series of strategies (described in theoretical and experimental works) to reduce the thermal conductivity of nanoscale channels are currently employed, i.e. the use of superlattice or superlattice-like geometries^15^,^16^,^17^,^18^, engineering the surface roughness^19^,^20^,^21^,^22^,^23^,^24^, the use of core-shell channels or channel coating^25^,^26^, twinning superlattice channels^27^, channel surface decoration and amorphization techniques, periodic channel width-modulation^28^,^29^,^30^,^31^,^32^,^33^.

Regarding the Si-Ge alloy, a stoichiometry of Si_0.8_Ge_0.2_ and crystalline orientation along (111) direction has been shown to provide the lower thermal conductivities and higher thermoelectric conversion efficiencies^2^,^11^. For bulk Silicon and Germanium, the room temperature thermal conductivities are ~140 W K^−1^m^−1^ and ~60 W K^−1^m^−1^ respectively^34^,^35^. However, Si-Ge alloys provide a significant reduction in thermal conductivity. Depending on the germanium content in silicon, values ranging from ~20 to ~9 W K^−1^m^−1^ can be achieved for bulk samples. The lowest value at room temperature thermal conductivity is achieved for an alloy with 20% germanium concentration in silicon (~9 W K^−1^m^−1^), as expected, which however, is still large for thermoelectric applications. In order to reduce the thermal conductivity on these structures even more, advanced materials engineering is desired, which could limit the transport of phonons more effectively. Some examples consist of nanostructuring bulk samples with still high level of crystallinity, which largely increases phonon-boundary scattering^36^, or the fabrication of multilayered films whose interfaces increase phonon scattering^37^, among others. Recent works in nanostructured Si_0.8_Ge_0.2_ films grown through Metal Induced Crystallization (MIC)^38^, have been able to achieve thermal conductivity values down to ~1.2 W K^−1^m^−1^ at room temperature. The reduction in thermal conductivity associated with the scattering alloying is ~9Wm^−1^K^−1^ for Si_0.8_Ge_0.2_^39^,^40^,^41^. Figure 1a shows a summary of the thermal conductivity of bulk (grey area) and nanostructures (square area) of silicon germanium as reported in the literature versus germanium content. This figure, based on previous theoretical and experimental studies on the Si-Ge alloys, shows that thermal conductivity of the alloy decreases drastically when the Ge concentration increases up to 20%, while it is approximately constant when the Ge concentration varies from 20% to 80%, exhibiting a U-shape dependence on Ge concentration^42^,^43^. This justifies the use of 20% Ge in most works that target thermal conductivity reduction. Figure 1b presents the state of the art values of the power factor achieved for Si_0.8_Ge_0.2_ structures^44^,^45^,^46^,^47^,^48^,^49^,^50^,^51^,^52^. For further phonon transport engineering, different technological strategies such as the fabrication multilayers and channel with reduced dimensionality such as nanotubes^53^ and nanowires^54^ have achieved significant reductions in the thermal conductivity.

A relatively new approach is to employ nano-meshes and vary the geometry of the mesh to alter the thermal conductivity. For that purpose, we fabricated pores in Si-Ge films as shown in Fig. 2. Silicon nano-meshed films with different pore diameters and highly ordered pore placement were grown previously using lithography process^55^. The lower thermal conductivity achieved was 1.73 W K^−1^m^−1^, corresponding to a silicon nano-mesh film with pore diameters of 55 nm. The effect of phonon confinement within the hollow structure and the coherent effects involved resulted in a thermal conductivity reduction of 99% in comparison to bulk silicon (~140 W K^−1^m^−1^). Theoretical works^56^,^57^ that study the effects of the porosity and roughness of silicon nano-meshed films also predict that the thermal conductivity of these structures can be significantly reduced in the presence of pores with roughened surfaces. However, the drawback of these films is the high cost and time consuming of the fabrication process. Moreover, since they are obtained in small areas, their thermal transport properties must be measured in specific microchips and might also present limitations in some applications.

In this work, Si_0.8_Ge_0.2_ nano-mesh films were grown via sputtering process on different diameter porous alumina substrates in large sizes of the order of cm^2^. During the sputtering growth, the Si_0.8_Ge_0.2_ films replicated the geometry of the porous alumina, giving rise to nano-meshed films in a quick, simple, and cheap way in comparison to other techniques^55^. Importantly, these matrices present high order and stability making them easy to handle. Thermal conductivities of 1.54 ± 0.27 W K^−1^m^−1^, 0.93 ± 0.15 W K^−1^m^−1^ and the ultra-low 0.55 ± 0.10 W K^−1^m^−1^ were found for our nano-meshed films with porous diameters of 294 ± 5 nm, 162 ± 11 nm and 31 ± 4 nm, respectively. Consequently, the geometry-dependent variation of the thermal conductivity observed for these nano-meshed films, opens the door for thermal engineering of these structures to achieve pre-specified thermal conductivities.

## Sample fabrication and measurement techniques

**Figure 2:**
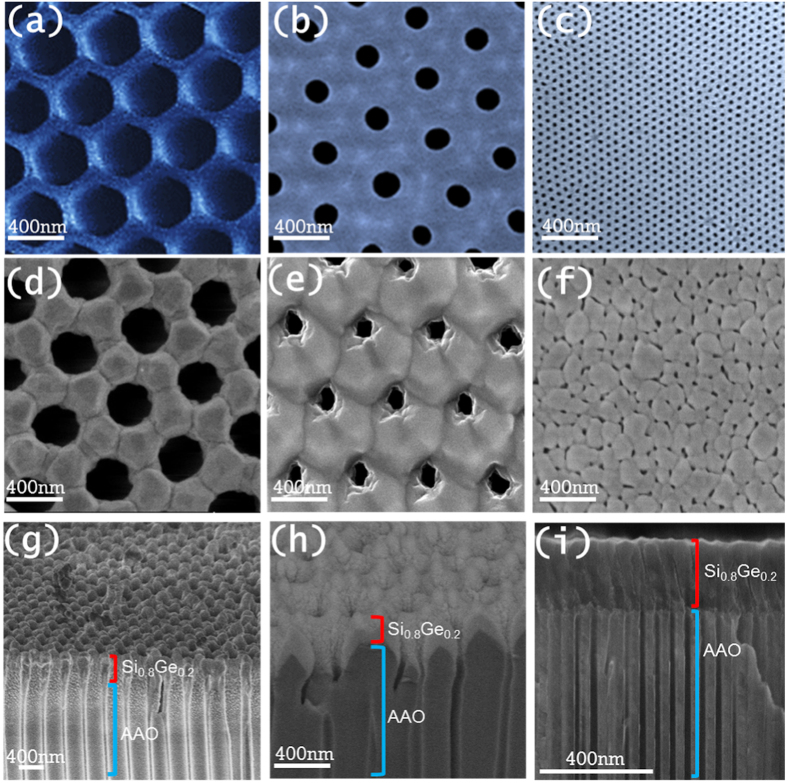
(**a–c**) are SEM images of porous alumina templates with 436 ± 16 nm, 162 ± 11 nm and 31 ± 4 nm diameters, respectively, that were used as substrates in the sputtering process. (**d–f**) show SEM images of the sputtered Si_0.8_Ge_0.2_ nano-meshed films grown on the previous templates, which have replicated the porous alumina. (**g–i**) are SEM images of the lateral of these samples, where the Si_0.8_Ge_0.2_ films and the alumina matrix can be observed.

In order to fabricate nano-meshed films, porous alumina matrices (AAO) highly oriented and with different pore diameters ranging from 436 ± 16 nm to 31 ± 4 nm were fabricated by different anodization procedures, as shown in refs 58 and 59 (see *Supporting Information*). Figure 2a–c show Scanning Electron Microscopy (SEM) images of the three different templates obtained, with 436 ± 16 nm, 162 ± 11 nm and 31 ± 4 nm diameters respectively. Then, these templates were used as substrates during the sputtering process, whose growing conditions are explained below in *Methods section*. The sputtered Si_0.8_Ge_0.2_ films replicated the porous structure of the alumina, resulting in the nano-meshed films with different pore sizes. This fabrication process allows growing large areas of Si_0.8_Ge_0.2_ nano-meshed films in a simple and reliable way, and can be easily industrially scalable. Figure 2d–f show the top view of the nano-meshed films that have replicated the pores of the alumina. In Fig. 2g–i the cross section view of these structures can be observed. The thickness of our Si_0.8_Ge_0.2_ films can be controlled depending on the time of the deposit (10 Å/s). Even for large thicknesses, ~3 μm, the replication of the pore structure is conserved. The porosity, diameter and distance between porous are summarized in Table 1 for both the alumina matrices and the nano-meshed films. (In Table 1, the smallest porous size of the Si-Ge film is not shown. Probably there is a huge dispersion (as seen from SEM image) but it might be interesting to show at least an average value (~31 nm).) It can be observed that the pore diameter and the porosity of the Si_0.8_Ge_0.2_ nano-meshed is slightly reduced from the pristine alumina template (AAO). The reason is that during the growing process, the Si_0.8_Ge_0.2_ prefers to replicate the structure widening the alumina template a bit and so reducing the pore size. In all the templates prepared during this study, the porous geometry is conserved, and the Si_0.8_Ge_0.2_ pores do not collapse.

**Figure 3:**
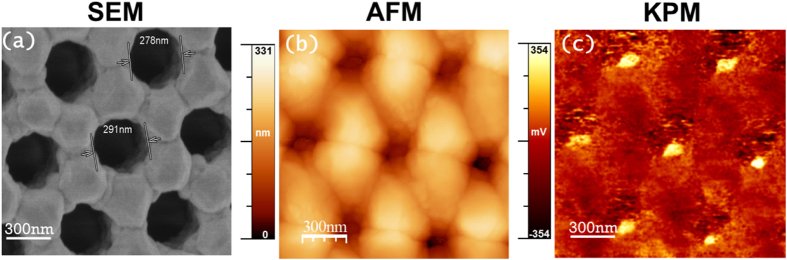
(**a**) SEM image of a Si_0.8_Ge_0.2_ nano-meshed film of ~294 nm porous size. (**b**) Topography by AFM And (**c**) surface potential image by KPM. The uniformity in the contrast of the KPM image reveals homogeneity in the surface potential of the film.

All nano-meshed Si-Ge films were oriented along the [111] direction, as revealed from X-Ray measurements (see *Supporting Information*). Raman spectra showed the three characteristic vibrational modes obtained for polycrystalline Si-Ge. The composition of these films was studied by X-Ray Photoemission Spectroscopy (XPS), resulting in the optimum stoichiometry of Si_0.8_Ge_0.2_ for all the samples. Moreover, an in-depth profile of the nano-meshed films, showed a small migration of oxygen from the alumina to the film, resulting in less of 7% of oxygen content in the film. The structure of the Si_0.8_Ge_0.2_ is zinc-blende^60^. The micro-Raman spectroscopy shows a homogenous phase in all the film (see *Supporting Information*). Additionally, the surface potential of the Si_0.8_Ge_0.2_ nano-meshed films was studied by Kelvin Probe Microscopy (KPM) (see *Supporting information*). Figure 3 shows the image for 294 ± 5 nm porous size nano-meshed film, which presents a homogeneous profile of the surface potential. It indicates that the work function of the films is homogeneous (confirming the homogeneity in the chemical composition obtained by Raman) and no potential drop is observed at the grain boundaries.

The fundamental thermal transport properties of the Si-Ge nano-meshed films were measured with three different setups. We measure the *out-of-plane* thermal conductivity at room temperature using a Scanning Thermal Microscope (*SThM*) working in *3ω-*mode, as shown in ref. 61. In this method, a thermo-resistive probe is brought into contact to the surface of the sample. When the probe contacts the sample, a heat flux flows through it. Depending on the thermal conductivity of the sample, the heat flux rate will be different, and so the temperature of the probe. As it is a thermo-resistive element, variations in the temperature of the probe involve changes in the electrical resistance of the probe. The third harmonic of the voltage response of the probe, V_3ω_, can be correlated with the thermal properties of the sample under study, as shown in ref. 61 This technique has been successfully used to measure the thermal conductivity of nano-structures, such as films^61^,^62^ and nanowires^63^,^64^,^65^. For that purpose, a thermo-resistive probe called Wollaston was set on the head of a Nanotec^®^ AFM system, which was used to position the probe on top of the samples^61^. Then, the third harmonic voltage response of the probe (V_3ω_) at low frequencies (10 Hz) was recorded with a lock in amplifier from Zurich Instruments^®^. Using this voltage signal, and after a proper calibration of the probe, the thermal conductivity of the sample can be calculated, as shown in ref. 61. The Supporting information summarizes the parameters and conditions used to measure these films.

Next, the *in-plane* electrical conductivity and Seebeck coefficient of both continuous and nano-meshed films prepared under the same conditions were measured with a home-built four probe system at room temperature, which has been previously successfully used to measure other samples (see *Supporting Information*). The homemade system consists of four electrical probes and two Peltier modules at the bottom. The electrical conductivity of the nano-meshed was carried out using the four probes of the system and the Van der Pauw method^66^. The Seebeck coefficient was measured by applying a temperature difference along the sample while measuring the voltage drop with two probes.

## Results and Discussion

**Figure 4:**
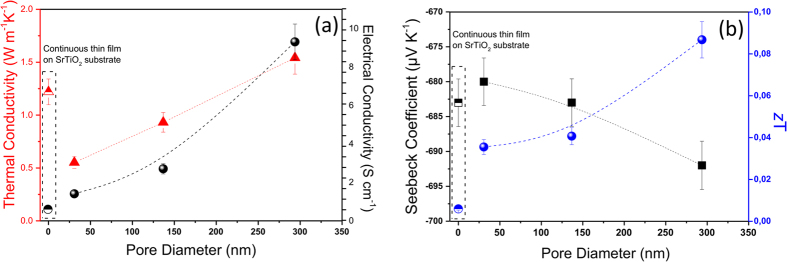
(**a**) Thermal conductivity (*κ*, red triangles) and electrical conductivity (*σ,* black spheres) and (**b**) Seebeck coefficient (*S*, black squares) and figure of merit (*zT*, blue spheres) plotted versus the pore diameter of the nano-mesh. The transport properties results obtained for a Si_0.8_Ge_0.2_ continuous thin film on SrTiO_3_ substrate grown under the same conditions are also plotted (the bullets/shapes inside the dotted rectangle on the left of each graph).

Figure 4a (left axis) shows the thermal conductivity results obtained at room temperature for the nano-meshed films with pore diameters ranging from 294 ± 5 nm to 31 ± 4 nm. The thermal conductivity reduction of the Si_0.8_Ge_0.2_ nano-meshed films varies from 1.54 ± 0.27 W K^−1^m^−1^ for the 294 ± 5 nm nanopore film, down to 0.55 ± 0.10 W K^−1^m^−1^ for the 31 ± 4 nm nanopore film. This figure reveals that the thermal conductivity of the nano-meshes can be engineered by the diameter of the pores (or alternatively the distance between the pores–and can be controlled in a linear fashion), while still keeping a higher order of crystallinity. At the left side of the figure, inside the dashed box, for comparison we show the thermal conductivity of a polycrystalline Si_0.8_Ge_0.2_ film without pores, grown under the same conditions as the nanomeshes, but on a SrTiO_3_ substrate (rather than the alumina template). The thermal conductivity of this polycrystalline Si_0.8_Ge_0.2_ film is 1.22 ± 0.21 W K^−1^m^−1^ (slightly lower than the nano-meshed material with 294 ± 5 nm nanopore diameter). This is not completely surprising as the continuous film is grown on a different substrate so it will present slightly different nanocrystals sizes than the nano-meshed film due to the difference in the way its nucleates, for example, but it provides an indication that large diameter holes (distanced further away from each other) might not affect the thermal conductivity significantly, but as the diameter (and the pitch) is reduced, this property is reduced significantly.

In order to explain the dependence of the thermal conductivity of our Si_0.8_Ge_0.2_ nano-meshed films with the diameter of the pores, the different phonon scattering mechanisms that play a role in these structures need to be considered. In ref. 56 it was observed that the thermal conductivity of silicon nano-meshed films can suffer a strong reduction in comparison to bulk silicon. The phonon scattering mechanisms occurring in these films were studied considering Monte Carlo simulations, including Umklapp scattering, boundary scattering, and pore scattering. While the contribution of the roughness to this reduction is low, the influence of the porosity and the placement of the porous contribute largely to this reduction. In another work, Tang *et al*.^55^ observed experimentally a reduction in thermal conductivity of silicon nano-meshed films when the diameter of the porous became smaller. The thermal conductivity was observed to depend on the small distances of the porous, which affect the mean free path of the phonons, the surface phonon scattering and a possible necking effect, which refers to phonons trapped in the holes/pores of the nano-meshed films that contribute to reduce the thermal conductivity. The authors showed that this last effect becomes more important as the diameter of the pore reduces.

Based on the previous theories and observations, the variation of the thermal conductivity of Si_0.8_Ge_0.2_ nano-meshed films versus the diameter of the pores, which is shown in Fig. 4, can be studied considering scattering effects similar to those occurring in the silicon nano-meshed films. For that purpose, in order to provide a first order understanding of phonon transport and the exceptionally-low thermal conductivity measured in the nano-meshes, we carried out thermal conductivity simulations based on the Callaway model theory. Using the Callaway model, the thermal conductivity is computed as follows:
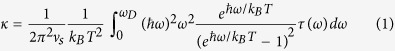
where *v*_*s*_is the sound velocity of the material, and τ(ω) is the phonon energy dependent relaxation time, for which in our case we include the effects of Umklapp three-phonon scattering τ_U_, boundary scattering on the top-bottom interfaces of the nano-mesh τ_b_, alloy scattering τ_a_, scattering by the crystallite boundaries τ_d_, and scattering by the pores τ_p_. These are connected using Matthiensen’s rule for a single relaxation time. We employ the usual formalism for all of the above mechanisms, i.e.:
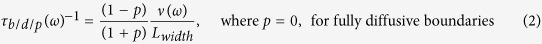

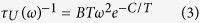

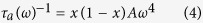
Above, *L*_width_ is the width of the material, or in general the distance between scattering interfaces, *x* is the Si fraction in the Si-Ge alloy, *B*, and *C* are numerical parameters used to fit the bulk material thermal conductivity^67^,^68^,^69^, and *A* is analytically determined from the usual alloy/impurity atoms scattering model^70^,^71^,^72^. For the sound velocity, as well as the parameters in the scattering mechanisms, we average the parameters used in literature for Si and Ge according to the alloy composition.

First of all, we evaluated the thermal conductivity of bulk Si and bulk Germanium at room temperature as ~140 W K^−1^m^−1^ and ~60 W K^−1^m^−1^, respectively. We then calculated the thermal conductivity of the Si_*x*_Ge_1-*x*_ alloy, and validate the values we obtain with the literature values for Si_x_Ge_1-x_ alloys of different compositions, especially for *x* = 0.8, which is the composition of the nano-meshes under consideration. For this calculation we included the effect of alloy scattering and phonon-phonon scattering (3-phonon Umklapp processes). Our results for *x* = 0.8 gives *κ* ~ 9 W/mK, which is approximately what the literature agrees on for Si_0.8_Ge_0.2_. To describe the thermal conductivity of the initial film, without the pores, we include in addition the effect of phonon scattering off the boundaries on the top and bottom of the film and the effect of phonon scattering on the boundaries of the nanocrystallites that form within the film (which form domains of ~70 nm in side lengths). In describing nanocrystalline domain boundary scattering we employ the same model as boundary scattering shown above. After this, our calculations indicate that the thermal conductivity drops to values *κ* ~ 1.7 W K^−1^m^−1^. Note that in the case of boundary scattering, we assume fully diffusive boundary scattering with *p* = 0, an approximation commonly employed in the literature to describe the boundary scattering in nanostructures^73^,^74^. This calculated value is slightly higher than that of the largest pore diameter nano-meshed film (294 nm, *κ* ~ 1.54 ± 0.27 W K^−1^m^−1^). On the other hand, the thermal conductivity of the continuous film on SrTiO_3_ substrate (Fig. 4a, left box), continuous film with no pores, is *κ* ~ 1.22 ± 0.21 W K^−1^m^−1^, which is somewhat lower compared to our calculations. Various reasons could be responsible for this, such as the presence of nucleation sites and clusters, and grain sizes. A more in detail information of the differences between the film grown on SrTiO_3_ and the mesh grown on amorphous gamma-alumina can be seen in the *Supporting Information*. In addition, the film contains ~7% oxygen doping, which could provide this further reduction in the thermal conductivity, possibly by introducing an additional alloy scattering mechanism, or by introducing changes in the phonon dispersion which reduce the sound velocity. Indeed, just by lowering the sound velocity of the material by ~10% in the simulations, the thermal conductivity drops to values *κ *~ 1.4 W K^−1^m^−1^, which resides within the error bars of the measured continuous films. Whether this oxygen alloy actually reduces the sound velocity in the material, or introduces further scattering (or both) to account for the reduction in *κ* is still under investigation, but it seems that at first order the effects of boundary scattering and alloying significantly reduce the thermal conductivity down to 1.7 W K^−1^m^−1^, and possibly oxygen presence, nucleation sites/clusters, and/or smaller grains, could provide another smaller, but still significant, reduction.

We next consider the influence of the nanopores on the thermal conductivity. In the calculations, scattering off the pores is again considered in a simplified manner as in the case of boundary scattering, *i.e.* by introducing an additional phonon randomizing scattering mechanism with characteristic length the distance between the pores. In the case of nano-meshed films with pore diameters of 294 ± 5 nm and 137 ± 8 nm, the calculations show that the thermal conductivity is indeed not reduced significantly, *i.e*. it is reduced to values *κ* ~ 1.3 W K^−1^m^−1^ and *κ* ~ 1.24 W K^−1^m^−1^, respectively. For the largest pore diameter nano-meshed film (294 nm), the measured thermal conductivity value is *κ* ~ 1.54 ± 0.27 W K^−1^m^−1^ and the calculated value (*κ* ~ 1.3 W K^−1^m^−1^) is within the measured error. Thus, both measurements and simulations indicate that the thermal conductivity is not much affected by the introduction of large diameter pores, despite the large porosity (~30%) with calculations suggesting that only a small reduction should be present. Note that the simulations used are based on models that take into account scattering in a simplified way, but still we are able to capture to a large extend the behavior of thermal conductivity in these films. More importantly, measurements and simulations are in agreement that more of the reduction of the thermal conductivity originates from alloying and boundary scattering, whereas the introduction of pores at least of large diameters does not alter this conclusion significantly.

As the pore diameter is reduced further the thermal conductivity drops (despite the fact that the actual porosity is less). For the nano-meshed films with pore diameter of 137 ± 8 nm, the measured value is κ ~ 0.9 W K^−1^m^−1^. This is lower compared to the calculated one (κ ~ 1.24 W K^−1^m^−1^), possibly due to more complicated effects that take place in the structure that are not captured by the simplified model we employ, or possibly due to coherent effects that reduce the sound velocity or introduce phononic bandgaps, that our semi classical model also does not capture.

Finally, as the pore diameter is reduced further, in the case of nano-meshed film with porous diameter of 31 ± 4 nm, measured thermal conductivity is κ ~ 0.55 ± 0.10 W K^−1^m^−1^, whereas the calculated thermal conductivity drops less to values κ ~ 0.9 W K^−1^m^−1,^ somewhat higher compared to the measured value. Considering the worst case scenario, *i.e*. the smaller feature sizes that were measured (rather than the average feature size) and that phonons scatter diffusively on boundaries defined by the smaller features (56 nm), the thermal conductivity drops to κ ~ 0.8 W K^−1^m^−1^, still somewhat higher compared to the measured values. This indicates that densely placed pores could introduce further disorder in the lattice (see Fig. 2f), or even coherent effects that reduce the sound velocity further, or even slight transport gaps that were observed in different cases^55^, which could account for this lower measured thermal conductivity and not captured by our simplified model.

In general, however, it is well described that most of the reduction in the thermal conductivity originates from the alloying and the grain-boundary scattering. The inclusion of oxygen has a smaller, but noticeable degrading effect. The introduction of pores with large feature sizes does not affect the thermal conductivity significantly, but as the feature sizes get smaller, a significant reduction can further be introduced. Still, however, in all cases the values of the thermal conductivity in both measurements and calculated data agree to be within κ ~ 0.5–1.5 W K^−1^m^−1^, a significant reduction with respect to the uniform Si_0.8_Ge_0.2_ alloy, which could be largely beneficial for thermoelectric applications since the power factor still remains considerable (see Fig. 2b). Finally, other scattering effects, such as necking effect^55^ or clusters formed in the several locations of the nano-meshes, might also be affecting the thermal conductivity, but were not considered here. We further note here that the structures we fabricated have some of the lowest thermal conductivities ever reported, at least for a Si-Ge based nanostructured system. Zhang *et al*. have reported slightly lower values (0.44 W K^−1^m^−1^) for a multilayer Sb_2_Te_3_/Au system^75^, whereas Chen *et al*. predicted by molecular dynamics simulations that a Si/Ge superlattice could also provide ultra-low thermal conductivities down to ~0.55 W K^−1^m^−1 ^42. In addition, Zhang *et al*. reported thermal conductivities of 0.5–1 W K^−1^m^−1^ in Bi/Bi_2_Te_3_ core-shell nanorods^76^. The nanomeshes presented in this work, however, provide the flexibility of cost effective fabrication of a large area material, which could be interesting for large scale applications.

Beyond the thermal conductivity, the rest of the thermoelectric coefficients, *i.e.* the electrical conductivities and the Seebeck coefficients of the nano-meshes versus the diameter of the pores, are shown in Fig. 4a,b. The electrical conductivity is larger for the larger diameter nano-meshes (even larger than that of the uniform film), and it reduces as the diameter of the pore becomes smaller by up to even an order of magnitude, approaching that of the uniform film (Fig. 4a-right axis). The reason for such behavior could be correlated to the fact that as the diameter of the pore becomes smaller the separation between pores also does (from 513 to 61 nm), as shown in Table 1, which could degrade the electronic transport. Nevertheless, the Seebeck coefficient shown in Fig. 4b (left axis) remains practically unaltered with values around −685 μV K^−1^. The power factors of the Si_0.8_Ge_0.2_ nano-meshed films varied from ~445 μW m^−1^ K^−2^ to ~65 μW m^−1^ K^−2^ at room temperature for the largest diameter pore nano-mesh (294 ± 5 nm) and the smallest one (31 ± 4 nm), respectively. Moreover, as the diameter of the pores reduces (Table 1), the power factor becomes more similar to the one obtained for the continuous film, ~24 μW m^−1^ K^−2 ^38. As the diameter of the pores is reduced, the porosity also reduces, and the structure starts to look more like a continuous film (with an additional scattering mechanism introduced by the pores). Moreover, it is worth mentioning that all these power factors are similar to those obtained for bulk Si_0.8_Ge_0.2_, reported in ref. 77. Finally, the values of the Figure of Merit obtained for these nano-meshes were plotted in Fig. 4b (right axis). These values are up to ~0.08 at room temperature for the nano-meshes with the larger pore diameters, which can be very useful for any industrial applications that work under this condition (room temperatures) and that require large sample areas based on an cost-effective materials and processes. Nevertheless, as explained above, the thermal conductivity is still much lower for the smaller pore diameter structures, as they are affected strongly by the scattering mechanics due to the presence of the small pores. We believe that these structures could be even more useful to thermoelectric and heat management applications once they are optimized to improve their electrical conductivity. Please also note that the *z*T value is just an estimation, since the power factor have been measured *in-plane* and the thermal conductivity in *out-of-plane* configuration, so we can only estimate the *z*T value. This assumption is only valid if this film has the same isotropic behaviors that the bulk Si-Ge alloy. In any case, we expect the in-plane thermal conductivity to be even lower, as phonons need to travel around the holes in the nanomesh.

We note that in a previous experimental study for Si nanomeshes^55^, *z*T ~0.4 at room temperature has been achieved with thermal conductivity ~1.73 W K^−1^m^−1^. In this work, however, the highest *z*T achieved is less than 0.1 at room temperature, resulting from the fact that the thermoelectric power factor in our system is lower. The lower power factor in our samples originates from a low electrical conductivity and a further reduction is observed as the pore diameter is reduced. While the Seebeck coefficient remains more or less unchanged and at relatively high values compared to literature. Higher power factors can be found for example in Neophytou^78^. However, it was not our intention to optimize the electrical conductivity, in this work we focused on reducing the thermal conductivity, which is significantly reduced further down to 0.55 W K^−1^m^−1^. Since high power factors have been previously achieved in such systems, one could think of combining the two methods (low thermal conductivity and high power factor) and achieve higher *z*T.

## Conclusions

Large area Si_0.8_Ge_0.2_ nano-meshed films with different porous sizes were fabricated through sputtering processes using alumina matrices as templates. This provides a novel approach to grow this kind of structures in a simple and reliable way. A large reduction in the thermal conductivity was observed due to alloying, and phonon boundary scattering on the upper/lower boundaries and crystallite boundaries within the nano-meshes. This is well explained within the Callaway model assuming fully diffusive boundary scattering. The thermal conductivity additionally drops with the introduction of pores, and seems to depend on the pore diameter and the distance between the pores. The smaller the pore diameter is, the larger the thermal conductivity reduction of the Si_0.8_Ge_0.2_ nano-mesh, due to enhanced scattering on the pore boundaries and due to possibly enhanced disorder or even coherent phonon effects that could be introduced. Using this approach, it is possible to control thermal transport of these films through nano-engineering. On the other hand, the nano-meshed power factors are larger in the structures with large pores (and larger distances between pores) rather than the more disordered structures which include denser pores with smaller diameters. The power factors are found to be between 450 μW K^−2^m^−1^ and 65μW K^−2^m^−1^, respectively, which seem to be as large as some of the last reported values for bulk Si_0.8_Ge_0.2_. This is attributed to the fact that the electrical conductivity in the nanomeshes with large inter-pore distance is much larger compared to the denser nanomeshes, whereas the Seebeck coefficient remains almost the same. Nevertheless, it is remarkable that the thermal conductivity in the small pore structures can be reduced to such low values (well below the amorphous limit in some cases), while still retain reasonable power factors, which opens the door for higher efficiency thermoelectric applications for this alloy once it is further optimized to improve its electrical conductivity.

## Materials and Methods

### Fabrication of highly ordered anodic aluminium oxide templates

The highly ordered hexagonal pore arrays throughout porous anodic alumina templates have been achieved by using simple two-step anodization^58^. Aluminum foils (99.999% purity, 0.5 mm thickness) supplied by Advent Research Materials (England) were first electropolished in perchloric acid/ethanol solution with a volume ratio of 1:4 for 4 min at 20 V after the cleaning and degreasing process. The first of the two anodization processes was applied to 6 h with constant voltage of 205 V at 4 °C in 1 wt% H_3_PO_4_ and 0.01 M aluminum oxalate (Alox) as electrolyte. The Alox is used as an additive to suppress breakdown of porous anodic alumina in the electrolyte of phosphoric acid at high potentials and comparatively high temperatures^59^. The second anodization was then performed under the same conditions as that of the first anodization after removing the disordered alumina film using the solution of chromic acid and phosphoric acid. The length of nanocavities can vary from hundreds nanometers to hundreds of microns and is controlled simply by the time of the second anodization process. In this case the time used was 12 h. Finally, a third step was carried out in order to widening the pores up to 350 nm in pore diameter. It consists in a controlled reduction of pore walls with a phosphoric acid solution, 5 wt% at 35 °C during 3 h. For the smaller diameter pores (25–30 nm) the first and the second anodization were performed in 0.3 M H_2_SO_4_, 25 V, 24 h, 1–2 °C.

#### Material growth and Characterization Methods

Silicon Germanium thin films were grown in a lab-designed sputtering system with a base vacuum of 10^−9^ mbar. A boron doped Si_0.8_Ge_0.2_ target (99.999% purity) was bonded onto a cylindrical (4′′) magnetron cathode in a vertical configuration. The growth chamber was evacuated to a base pressure of 5 × 10^−9^ mbar by turbo pumping, using ultrapure argon (99.9999%) as the sputtering atmosphere. DC plasma was activated with a voltage of 720V and 80mA at a pressure of 7 × 10^−3^ mbar. For the thermal treatment a lab-made substrate heater holder was designed, which can reach temperatures up to 750 °C. The temperature was controlled by means of a *EUROTHERM* 3216 controller and the temperature was measured by a type K thermocouple attached to the center of the sample holder surface. The crystalline structure of the films was studied by X-ray diffraction (*XRD*) using a *Philips* X-PERT diffractometer with a Cu K_*α*_ radiation source with a wavelength of 1.54 ± 0.2718 Å in Bragg-Brentano geometry. Diffraction patterns were identified by standard reference patterns, supplied by the International Centre for Diffraction Data (ICDD). Micro-Raman spectrometer (*Horiba Jobin Yvon*) LabRam HR with a 532 nm Nd:YAG laser (8.5 mW) was used for compositional mapping and local crystallization. Scanning electron microscopy has been performed on a *JEOL* JSM-6460LV and the *AFM* images were obtained with a *Nanotec*^®^ *AFM* microscope. *XPS* spectra were recorded on a custom *Specs* X-ray Photoelectron Spectroscopy system (Hemispherical Energy Analyzer PHOIBOS 100/150). Monochromatic Al K_*α*_ (E = 1486.6 eV) emission was used as the X-ray source. The pressure of the analysis chamber was kept at 10^−10^ mbar. Survey *XPS* spectra and narrow (for recording high resolution peaks) scan *XPS* spectra were collected with pass energies of 0.5 eV and 0.02 eV, respectively. The analyzed area was approximately 1.4 mm^2^. Peak analysis was performed by using Gaussian-Lorentzian convoluted bands, and a Shirley non-linear sigmoid-type baseline. All spectra were calibrated with the C1s peak located at 284.8 eV to set the binding energy scale. The data were analyzed with CasaXPS^®^ software (*CasaSoftware* Ltd). Resistivity measurements and Seebeck coefficient were measured with a home-made four probe system, which was used to cross-check the data obtained from a commercial *Linseis*^®^ LSR-3 system from room-temperature until 500 °C. Thermal conductivity measurements were carried out with a Scanning Thermal Microscope (SThM) working in 3ω mode^61^. More details of the measuring procedure are given in supporting information.

## Additional Information

**How to cite this article**: Perez-Taborda, J. A. *et al*. Ultra-low thermal conductivities in large-area Si-Ge nanomeshes for thermoelectric applications. *Sci. Rep.* **6**, 32778; doi: 10.1038/srep32778 (2016).

## Supplementary Material

Supplementary Information

## Figures and Tables

**Figure 1:**
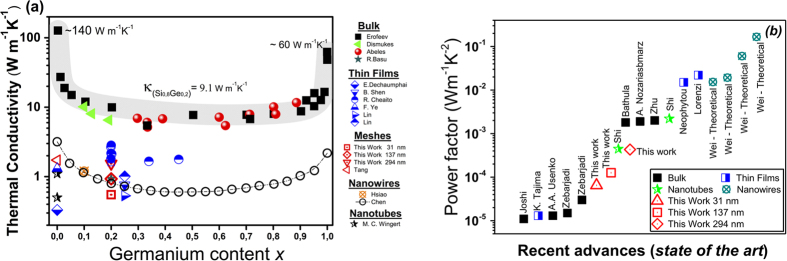
Literature reported (**a**) thermal conductivity values for Si-Ge structures versus germanium content^36^,^37^,^39^,^40^,^41^,^43^,^53^,^54^,^80^,^81^,^82^ and (**b**) the state of the art of the power factor for Si_0.8_Ge_0.2_ structures.

**Table 1:** Summary of the geometrical properties observed for both the pristine alumina templates and the Si_0.8_Ge_0.2_ nano-meshed films grown on top.

Sample	Pore Diameter (nm)	Pore distance (nm)	Porosity %	Roughness (nm)
(a) AAO	436 ± 16	508 ± 22	51 ± 2	95 ± 8
(b) AAO	162 ± 11	480 ± 16	11 ± 1	75 ± 9
(c) AAO	31 ± 4	61 ± 1	13 ± 2	15 ± 4
Si-Ge Film	0	0	0	5.2 ± 2
(d) Si-Ge	294 ± 5	513 ± 8	30 ± 2	53.3 ± 7
(e) Si-Ge	137 ± 8	477 ± 16	5 ± 1	35 ± 3
(f) Si-Ge	19 ± 11	55 ± 13	3 ± 1	15 ± 4

The Si-Ge film has been added for comparison purposes.
