# Supplementary material for: Ultra-low thermal conductivities in large-area Si-Ge nanomeshes for thermoelectric applications
Source: Sci Rep. 2016 Sep 21;6:32778. doi: 10.1038/srep32778 (PMC5030677; doi:10.1038/srep32778)
Supplement: Supplementary Information [file srep32778-s1.pdf]

SUPPORTING INFORMATION:

Ultra-low thermal conductivities in large-area SiGe nanomeshes for thermoelectric applications

Jaime Andrés Pérez-Taborda[†], Miguel Muñoz-Rojo[†], Jon Maiz[†‡], Neophytos Neophytou[§], Marisol Martín-González[†]*

[†] Instituto de Microelectrónica de Madrid (IMM-CSIC), Calle de Isaac Newton 8, Tres Cantos, 28760 Madrid, Spain, [§]School of Engineering, University of Warwick, Coventry, CV4 7AL, UK

KEYWORDS: Nano-meshes, Silicon Germanium, Thermoelectric Materials, DC – Sputtering, Thermal Conductivity

S1. Structural characterization techniques

The crystalline structure of the films was studied by X-ray diffraction (*XRD*) using a *Philips* X-PERT diffractometer with a Cu K_{α} radiation source with a wavelength of 1.5418 Å in Bragg-Brentano geometry. Diffraction patterns were identified by standard reference patterns, supplied by the International Centre for Diffraction Data (ICDD). Micro-Raman spectrometer (*Horiba Jobin Yvon*) LabRam HR with a 532 nm Nd:YAG laser (8.5 mW) was used for compositional mapping and local crystallization. Scanning electron microscopy has been performed on a *JEOL* JSM-6460LV and the *AFM* images were obtained with a *Nanotec AFM* microscope. Resistivity measurements and Seebeck coefficient were measured with a commercial *Linseis* LSR-3 system.

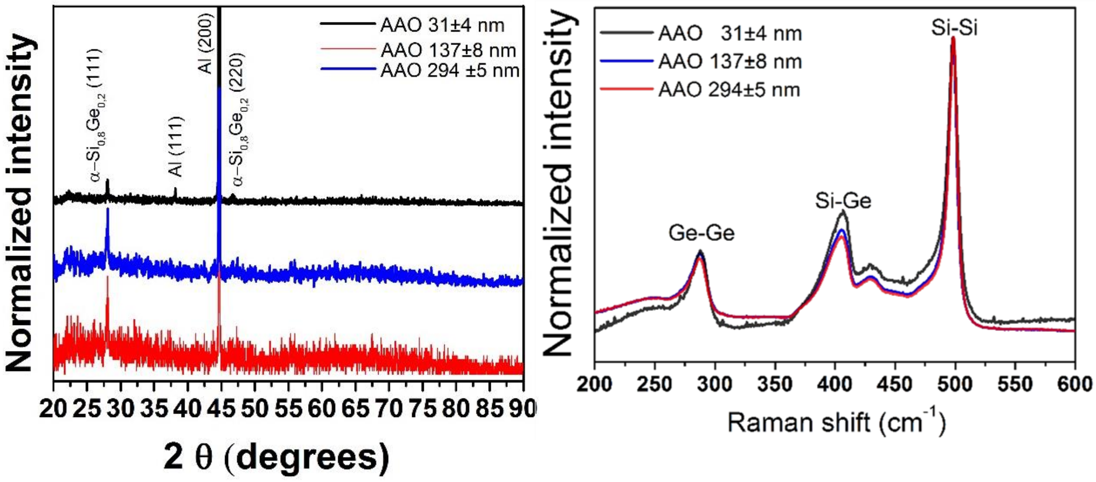

Figure S1. a) X-Ray and b) Raman spectra of a $Si_{0.8}Ge_{0.2}$ grown on nano-meshes with pore diameter of 31 nm (black line) 137 nm (blue line) and 294 nm (red line).

Substrate	[111] $Si_{0.8}Ge_{0.2}$ 2θ (º)	Peak Width (degrees)	Approx. Crystallite size
AAO 25nm	28,0608	0,1692	*≈ 50 nm*
AAO 137nm	28,07	0,1342	*≈ 65 nm*
AAO 294nm	28,0474	0,2497	*≈ 35 nm*
$SrTiO_3$	28,0255	0,1235	*≈ 70 nm*

Table SI. Average $Si_{0.8}Ge_{0.2}$ crystallite size. They have been calculated from XRD data using the Scherrer equation with a copper Kα (λ = 1.54056 Å) and a constant of 0.94.

S2. Porous alumina fabrication.

The highly ordered hexagonal pore arrays throughout porous anodic alumina templates were achieved by using a two-step anodization.[2] Aluminum foils (99.999% purity, 0.5 mm thickness) supplied by Advent Research Materials (England) were first electropolished in perchloric acid/ethanol solution with a volume ratio of 1:4 for 4 min at 20 V after the cleaning and degreasing process. The first of the two anodization processes was applied to 6 h with constant voltage of 205 V at 4ºC in 1 wt% H_3PO_4 and 0.01 M aluminum oxalate (Alox) as electrolyte. The Alox is used as an additive to suppress breakdown of porous anodic alumina in the electrolyte of phosphoric acid at high potentials and comparatively high temperatures.[3] The second anodization was then performed under the same conditions as that of the first anodization after removing the disordered alumina film using the solution of chromic acid and phosphoric acid. The length of nanocavities can be varied from hundreds nanometers to hundreds of microns and it is controlled by the time of the second anodization process. In this case the time used was 12 h. Finally, a third step was carried out in order to widening the pores up to 350 nm in pore diameter. It consists in a controlled reduction of pore walls with a phosphoric acid solution, 5 wt% at 35ºC during 3 h.

The sputtering of the $Si_{0.8}Ge_{0.2}$ on top of these templates resulted in films that replicated the porous structure, i.e. nano-meshed $Si_{0.8}Ge_{0.2}$ films. Figure S2 shows an optical image of the porous alumina before and after depositing $Si_{0.8}Ge_{0.2}$ film on top.

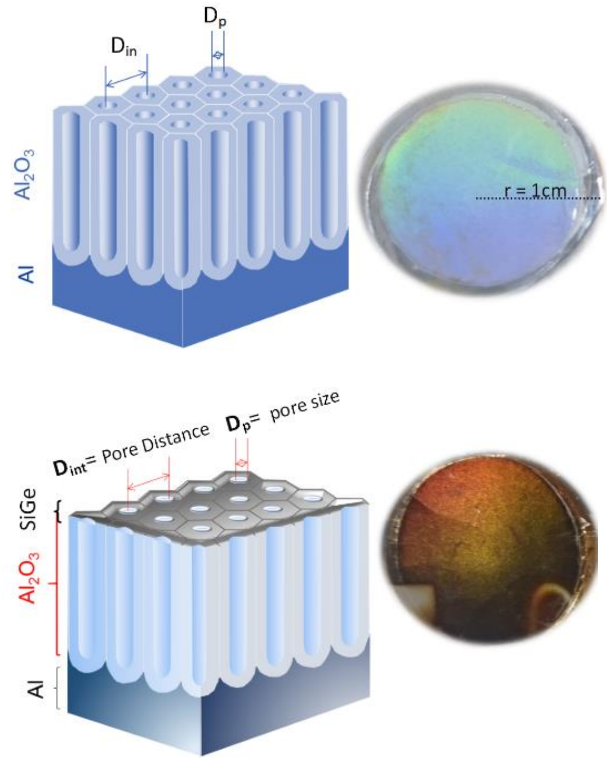

Figure S2. Sketch and optical image of a) a porous alumina template and b) the SiGe film nano-mesh.

S3. Continuous film fabrication.

A $Si_{0.8}Ge_{0.2}$ film without pores was grown through DC plasma sputtering system. The conditions used to fabricate were similar to those used for nano-meshes. A thickness of 400 nm and average roughness of 2,5 nm was observed from a profilemeter and an Atomic Force Microscopy (AFM) **Figure S3**. The structural and transport properties of these films are shown in reference[1].

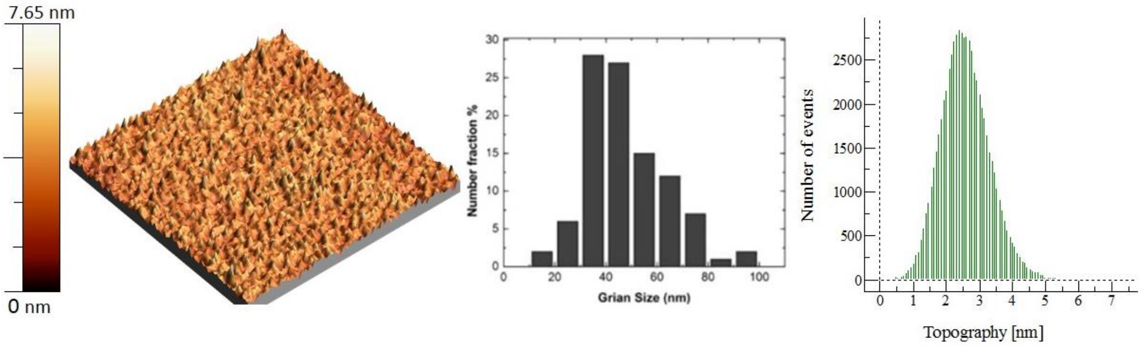

Figure S3. Roughness of the continuous thin film on $SrTiO_3$ substrate. The statistical average of the roughness is 2.5±0.3 nm.

S4. AFM and KPM analysis for the $Si_{0.8}Ge_{0.2}$ nano-meshed films.

Images of the topography of the porous $Si_{0.8}Ge_{0.2}$ nano-meshed films were taken with an atomic force microscope (AFM). The porous mean diameter was studied with this technique. Figures S4, S5 and S6 show the images obtained with the AFM.

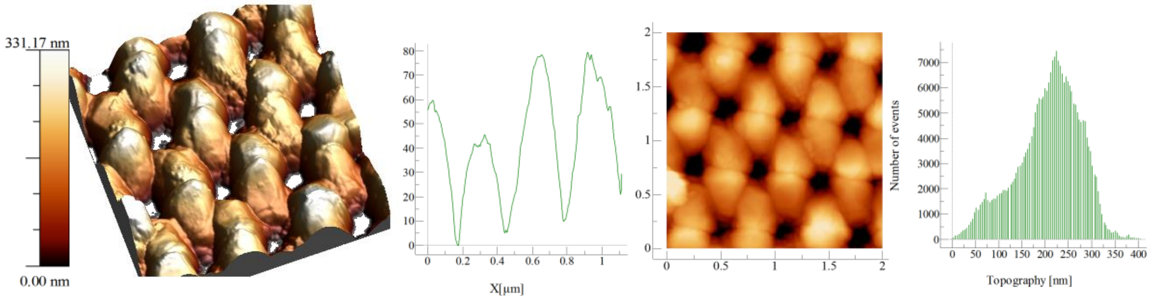

Figure S4. Roughness of the SiGe film deposited on the template alumina with pore size of 294±5 nm. The statistical average of the roughness is 53±7 nm.

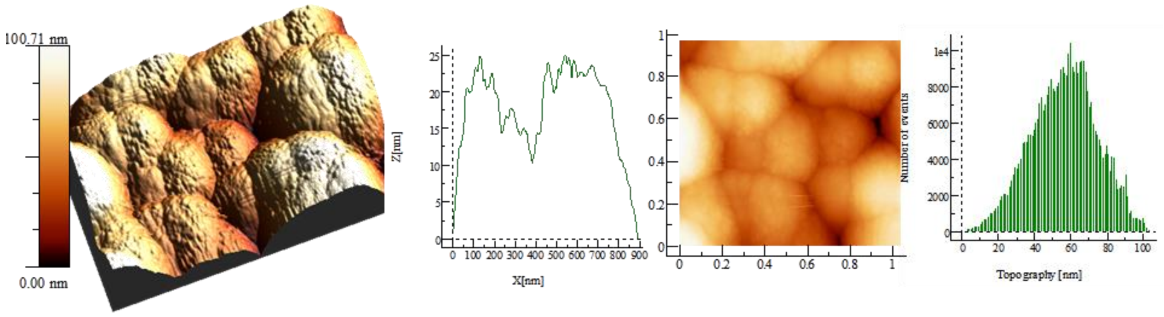

Figure S5. Roughness of the SiGe film deposited on the template alumina with pore size of 137±8 nm. The statistical average of the roughness is 14±8 nm.

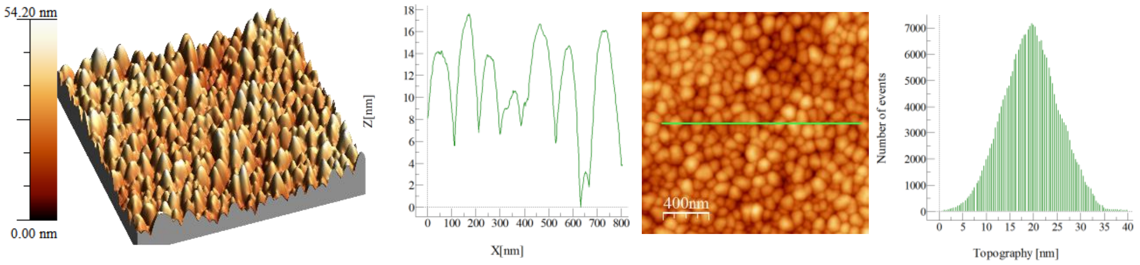

Figure S6. Roughness of the SiGe film deposited on the template alumina with pore size of 31±4 nm. The statistical average of the roughness is 5±2 nm.

Moreover, Kelvin Probe Microscopy (KPM) images were taken for the three nano-meshed films with pore diameter ranging from 294±5 nm to 31±4 nm diameter. Figure S7 show these pictures were homogeneous surface potential is observed for the samples.

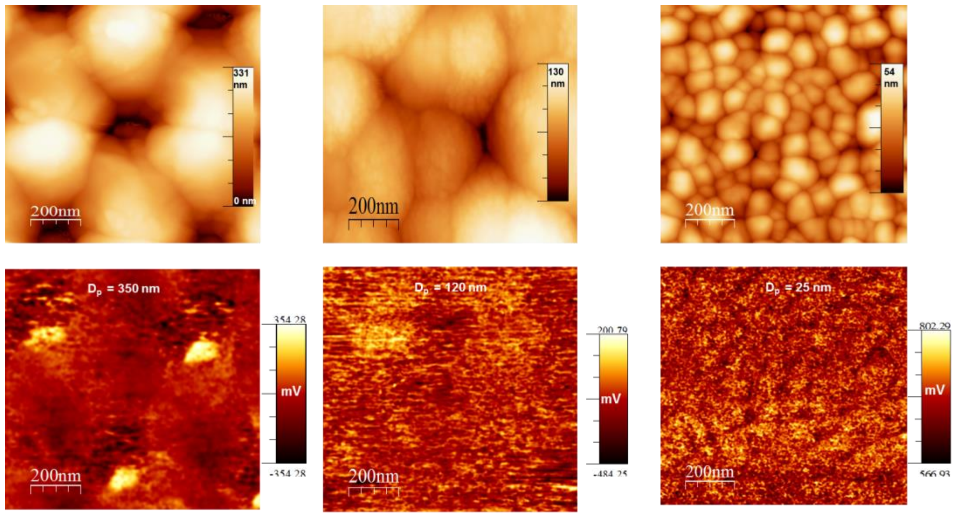

Figure S7. a),b) and c) are topographic images of the surface of nano-meshed films with pore diameters of 294±5 nm, 137±8 nm and 31±4 nm, respectively. d), e) and f) are the surface potential images of these films, which reveal an homogeneity in the material composition.

S8. Scanning Thermal Microscopy (SThM) in 3ω mode.

The thermal conductivity measurements of the SiGe nanomeshes were performed using the SThM working in 3ω mode, which has been successfully used to measure the thermal conductivity of films and nanowires [4,5-8]. For that purpose, an AFM system from Nanotec® Company was used to position a thermoresistive probe on top of the sample. The probes used in this case were Wollaston probes from Bruker® company. The measuring and analysis procedure was similar to those presented in reference [4].

Firstly, the probe needs to be calibrated[4] in order to obtain the thermal exchange radius, *b*, and the contact resistance, *Rc*, between the probe and the sample. Following the same procedure as shown in ref. [4], we measured a set of three samples with well-defined thermal conductivity. These calibration samples were polyaniline (PANI) with 5% and 7% graphene platelets and borosilicate glass with thermal conductivities of k=0.49 W/K·m, k=0.65 W/K·m and k=1.1 W/K·m, respectively. As in reference[4], Figure S8 shows the crossing between curves for the probe and calibration samples used, giving a value of *b*=(2.27±0.09) µm and *Rc*= 29365±6925 (K/W). The probe convection coefficient was determined to be, *h*=3324 W/K·m^2.

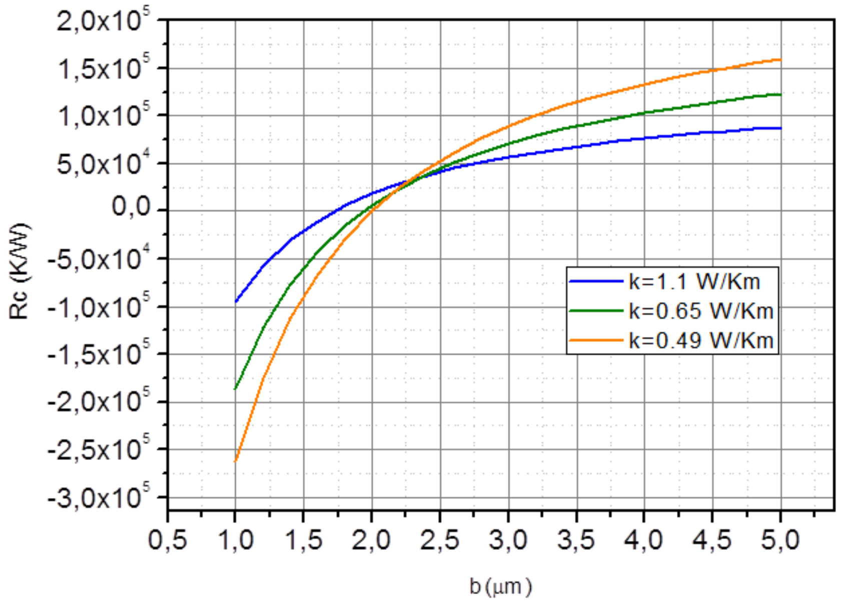

Figure S8. Thermal exchange radius vs contact resistance graph obtained during the calibration process for the Wollaston probe used.

After the calibration was carried out, the thermal resistance of our SiGe nanomeshed samples, with pore diameters of 294±5 nm, 137±8 nm and 31±4 nm were obtained with this technique in a similar way as explained in reference[4]. Due to its small film thickness (around 1µm or less), the heat flows across the SiGe film but also through the alumina substrate underneath the film. The thermal resistances of the 294±5 nm, 137±8 nm and 31±4 nm porous size diameter samples, whose values are influenced by the SiGe film, the air of the porous and the alumina substrate, were determined to be 124829 K/W, 127366 K/W and 165795 K/W, respectively. As a consequence, the semi-infinite theory to extract the thermal conductivity of the intrinsic SiGe film (R=1/4kb) cannot be used. Instead, 2D COMSOL® simulations were performed to determine the intrinsic thermal conductivity of this film [4].

We considered the geometry of the sample, in which the thickness of the SiGe film, the porosity and the size porous were considered. On top of the film, a Gaussian heat disc source with the same radius as the thermal exchange radius of the Wollaston probe was set. Regarding the material properties, the thermal conductivity of the alumina was measured with the photoacoustic technique resulting to be 1.33 W/Km while the one for the SiGe film is unknown. Therefore, the thermal conductivity of the SiGe nanomesh film will be varied until the thermal resistance obtained from the simulation, R_{simult}, matches with the thermal resistance obtained experimentally, R_{experm}. The symmetry of the sample/measurement is taken as an advantage in the simulation to speed it up[4].

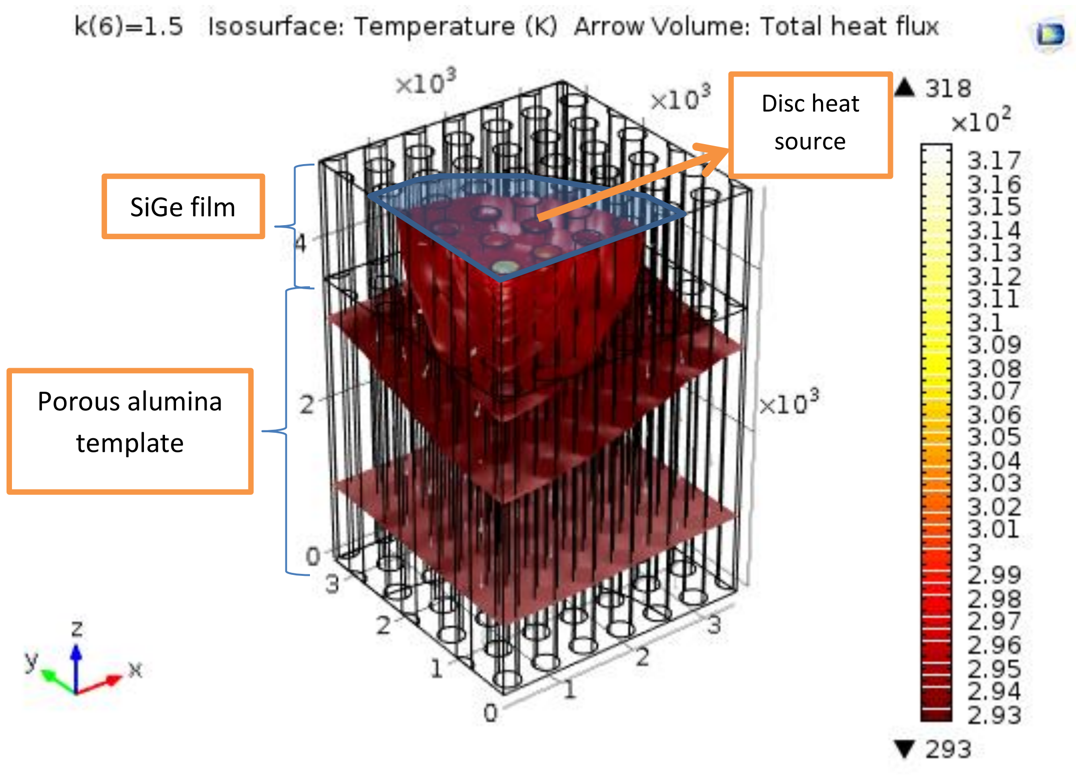

Figure S8a shows the isothermal contour obtained from the simulation of the 294±5 nm SiGe nanomesh. Figure S8b-d shows the comparison between the simulation (R_{simult}) and the experimental result (R_{experm}).

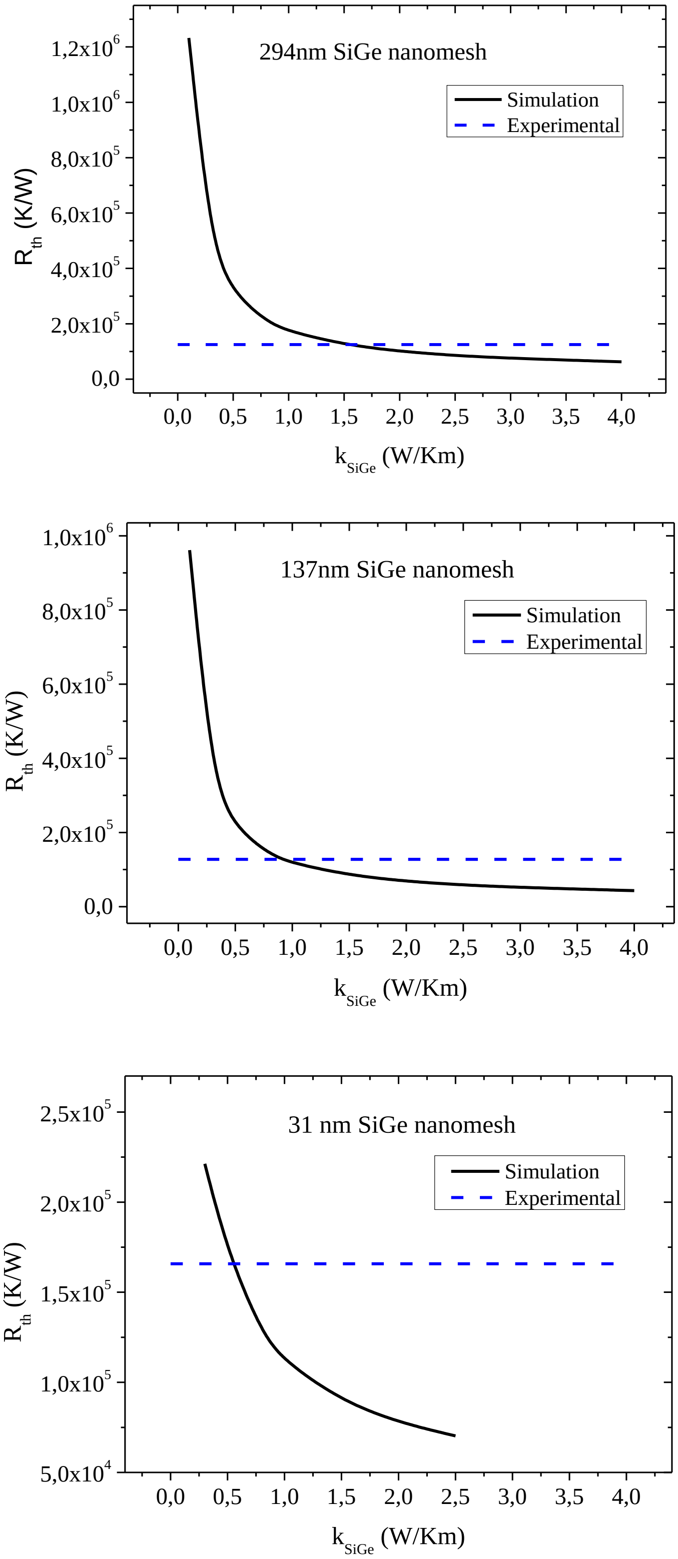

Figure S8. a) Simulation result for the 294 nm SiGe nanomesh. The SiGe film is on top of the poropus alumina substrate. b), c) and d) show the thermal resistances obtained from simulation (black line) and from the experiment (blue line). The matching point indicates the thermal conductivity of the SiGe film.

Table IIS summarizes the thermal conductivity results obtained for the 294 nm, 137 nm and 31 nm SiGe nanomeshes after the simulation was performed.

Table IIS. Thermal conductivities obtained for the SiGe nanomeshes.

SiGe nanomesh-porous size	Thermal Conductivity (W/Km)
**294±5 nm**	**1.54±0.27**
**137±8 nm**	**0.93±0.15**
**31±4 nm**	**0.55±0.10**

REFERENCES

1 Taborda, J. A. P. *et al.* Low thermal conductivity and improved thermoelectric performance of nanocrystalline silicon germanium films by sputtering. *Nanotechnology* **27**, 175401 (2016).

2 Masuda, H. & Fukuda, K. Ordered Metal Nanohole Arrays Made by a Two-Step Replication of Honeycomb Structures of Anodic Alumina. *Science* **268**, 1466-1468 (1995).

3 Sun, C., Luo, J., Wu, L. & Zhang, J. Self-ordered anodic alumina with continuously tunable pore intervals from 410 to 530 nm. *ACS Applied Materials and Interfaces* **2**, 1299-1302 (2010).

4 Wilson, A. *et al.* Thermal conductivity measurements of high and low thermal conductivity films using a scanning hot probe method in the 3[small omega] mode and novel calibration strategies. *Nanoscale* **7**, 15404-15412, doi:10.1039/C5NR03274A (2015).

5 Maiz, J. *et al.* Enhancement of thermoelectric efficiency of doped PCDTBT polymer films. *RSC Advances* **5**, 66687-66694 (2015).

6 Wilson, A. A. *et al.* Thermal conductivity measurements of high and low thermal conductivity films using a scanning hot probe method in the 3 ω mode and novel calibration strategies. *Nanoscale* **7**, 15404-15412 (2015).

7 Rojo, M. M. *et al.* Decrease in thermal conductivity in polymeric P3HT nanowires by size-reduction induced by crystal orientation: new approaches towards thermal transport engineering of organic materials. *Nanoscale* **6**, 7858-7865, doi:10.1039/C4NR00107A (2014).

8 Muñoz Rojo, M., Caballero Calero, O., Lopeandia, A. F., Rodriguez-Viejo, J. & Martin-Gonzalez, M. Review on measurement techniques of transport properties of nanowires. *Nanoscale* **5**, 11526-11544, doi:10.1039/C3NR03242F (2013).
